# The evaluation of performance for agroecological greenhouse tomato strategies by the CRITIC-OWA model

**DOI:** 10.3389/frai.2025.1599334

**Published:** 2025-06-18

**Authors:** José Manuel Brotons-Martínez, José María Cámara-Zapata

**Affiliations:** ^1^Economic and Financial Department, Miguel Hernández University, Elche, Spain; ^2^Institute for Agri-Food and Agro-Environmental Research and Innovation, CIAGRO, Miguel Hernández University, Orihuela, Spain

**Keywords:** economic criteria, social criteria, environmental criteria, pearson coefficient, agrovoltaic, photovoltaic energy, deficit irrigation

## Abstract

**Introduction:**

Modern agriculture must begin to use production strategies that are increasingly sustainable. To help in decision-making, the present work analyzes the sustainability of greenhouse tomato production with different agroecological strategies: shading (conventional fixed mesh and mobile photovoltaic shading), grafting and deficit irrigation, based on economic, social, and environmental criteria.

**Methods:**

For the ranking of the different strategies, the use of an extension of the CRiteria Importance Through Inter-criteria Correlation (CRITIC) is proposed, in which the correlation between the criteria is obtained through the Pearson-OWA, where the aggregation of the quadratic differences between criteria is carried out considering the attitudinal character of the decision-maker, that is, using Ordered Weighted Averaging (OWA), in addition to induced variables, with the Induced Probabilistic OWA CRITIC (IPOWA CRITIC). Three extensions are considered based on this model depending on the way the multicriteria score is calculated: i) the ranking is carried out on the relative score (S) of each alternative (IPOWA-S-CRITIC), ii) on the weighting vector (W) (IPOWA-W-CRITIC), or iii) on both (IPOWA-S-W-CRITIC).

**Results:**

The results of the classifications conducted indicate that the use of mobile photovoltaic mesh is a sustainable production strategy, due to its effect on production and quality of the crop, CO2 fixation, and irrigation water savings.

**Discussion:**

The use of mobile photovoltaic shades is compatible with tomato cultivation in a greenhouse if the management of the installation is performed considering the needs of the plants in most of the rankings.

## 1 Introduction

Presently, climate perturbations include extreme temperature values that increase the evapotranspiration of plants and irrigation needs, compromising agricultural production, especially of crops in Mediterranean climate areas, due to the scarce quantity and quality of the water available (Giordano et al., 2021). Tomatoes (*Solanum lycopersicum* L.) are the second most-cultivated crop in the world after potatoes, with approximately 187 million tons and 5 Mha in 2021, according to the statistical results from the Food and Agriculture Organization of the United Nations (FAOSTAT, 2025). This crop has high water and nutritional demands and is also sensitive to the reduction in photosynthesis due to photoinhibition, so it is strongly affected by climate change (Mutale-Joan et al., 2020).

In tomato cultivation, grafts are one of the most utilized agroecological techniques. This technique was originally used for controlling pathogens (Louws, 2012), and it is currently used to improve production and quality (Turhan et al., 2011), or to favor the crop's adaptation to conditions of abiotic stress, such as drought, salinity, and high temperatures (Kumar et al., 2017). Shading limits the effect of solar radiation on crops, reducing transpiration and the associated consumption of irrigation water and fertilizers, as well as the leaching of nutrients (Ghoulem et al., 2019). In addition, it improves the homogeneity of the climate and increases productivity and the quality of crops that are especially sensitive to photoinhibition, such as tomatoes (Briassoulis et al., 2007). Photovoltaic technologies directly convert sunlight into electrical energy, thanks to the photoelectric effect. Agrovoltaic applications in open-air crops and greenhouses have been investigated since the start of the 21^st^ century (Magadley et al., 2020). Specifically for tomato, the recommendation is for the modules not to exceed 20% of shading, and it is estimated that shading the entire surface of the crop reduces solar radiation by 80%, which leads to a decrease in production of 70% (Cossu et al., 2018; Kumar et al., 2022). In recent years, there has been a growing number of investigations for possible solutions that make short-term forecasts and identify an unrecognized evaluation standard (Moreno et al., 2025).

A cost–benefit study will allow farmers and growers to make advances in the optimization of agricultural production (Cámara-Zapata et al., 2019). However, assessing the sustainability of different agricultural production strategies requires a multicriteria hierarchical analysis, considering agronomic, economic, environmental, and social criteria (Brotons-Martínez et al., 2024). In this way, it is possible to rank different production strategies considering the result of this analysis. Among the methods utilized, the Monte Carlo analysis, the determination of accumulated probabilities, and polling experts, stand out. However, all of these lack the objectivity necessary to make a decision about the adequacy of the strategies analyzed. Thus, it is necessary to use new ranking methodologies that contribute toward consolidating the motivation of the decisions to be made.

The Criteria Importance Through Inter-Criteria Correlation (CRITIC) method was introduced by Diakoulaki et al. (1995). Its objective is to rank a set of alternatives based on a series of criteria. This method uses the information available and objectively assigns weights to the different criteria through an analytical investigation of the evaluation matrix, quantifying the intrinsic information of each assessment criterion through the value of its standard deviation and the relative discrepancy between the values of each criterion, measured through Pearson's correlation. According to Luo et al. (2024), the estimation of the indicator weight, based on the intensity of comparison, that is, the standard deviation, and the conflict between the evaluation indicators, is used as an objective assignment. To introduce the degree of optimism or pessimism of decision-makers, the combination of this method with a very common aggregation method, the ordered weighted averaging (OWA) operator introduced by Yager (1988), is proposed. The OWA operator considers an aggregation process, providing the maximum, the minimum, and the average. A generalization in the variance and the covariance, allowing for a wide range of scenarios from the minimum to the maximum, that is, from the most optimistic to the most pessimistic scenario, can be followed in Yager (1996a) and Merigó (2011). The OWA linear regression (LR) was introduced by Yager and Beliakov (2010). Flores-Sosa et al. (2020) present an application that uses simple linear regression and the Induced OWA operator in the same formulation.

The CRITIC method and the OWA operator and its extensions have been used in a wide range of applications (Peng and Huang, 2020; Diakoulaki et al., 1995; Merigó and Casanovas, 2011; Yager, 1996b). Although some published studies have combined both concepts (Luo et al., 2024; Xing et al., 2022), they have been presented as two independent methods. Some studies have introduced the OWA in aggregating relative scores (Brotons-Martínez et al., 2024). The CRITIC method has been proposed for several applications in agriculture, such as the evaluation of irrigation systems (Hezam et al., 2024), the selection of suitable reference evapotranspiration (ETo) models (Islam et al., 2020), or for selecting the best alternative for using reclaimed water in India (Narayanamoorthy et al., 2019). The main advantage of the CRITIC method is that it computes the conflict and variability of the criteria by calculating their weights objectively, by analyzing their variability and inter-correlation. The CRITIC method not only avoids the interference of subjective factors but also considers the contrast intensity and conflict between indicators to determine the weight (Anwar, 2021).

Some studies use CRITIC combined with other methodologies, such as the Technique for Order Preference by Similarity to Ideal Solution (TOPSIS) in multicriteria decision-making (Liu et al., 2024), the gray relational analysis (Xu et al., 2020; Mishra and Muhuri, 2021), the Analytic Hierarchy Process (AHP) (Zhao et al., 2022), the AHP combined with multicriteria optimization and compromise solution (VIKOR) (Feng et al., 2021), a gray multicriteria decision-making combined compromise solution (Yazdani et al., 2024), the qualitative flexible multiple criteria (QUALIFLEX) method (Liu et al., 2022), the Extended Distance from the Average Solution (EDAS) under a mixture Z-number environment (Sun et al., 2022), the entropy weight method (EWM) (Yuan et al., 2024), the neutrosophic linguistic MCDM (MultiCriteria Decision-Making) algorithm based Combined Compromise Solution (CoCoSo) (Peng and Huang, 2020), or with the Taxonomy method extended to the intuitionistic fuzzy numbers (Xiao et al., 2020). All of these studies deal with methodological combinations that try to improve the CRITIC method, but without considering the attitudinal characteristic of the decision-maker.

The aim of the study is to obtain a CRITIC method where the conflict and variability of the criteria could be considered for estimating the weights according to a degree of optimism to obtain different forecast scenarios. Moreover, by using the Induced OWA (IOWA) and Induced Probabilistic OWA (IPOWA) operators, the decision-maker can under- or over-estimate the information according to a complex attitude that includes the degree of optimism and the psychological and competitive factors (Flores-Sosa et al., 2020). Thus, using induced operators helps us work with complex variables for which the greatest benefit is not always the best solution. For example, this may occur depending on the results that competitors obtain or some personal opinions about the alternatives.

The main novelty of the study is the introduction of the OWA in the analysis of the correlation among different criteria. Using extensions such as the Pearson-POWA allows combining the attitudinal character with the probability of commercial implementation of each treatment. Finally, the Pearson-OWA makes it possible to assign the importance of each sum of the correlation coefficients as a function of an induced variable, the sum of the normalized values of the different criteria for each treatment.

The manuscript is structured in the following manner: first, the basic concepts of the OWA, variance-OWA, and covariance-OWA are described. Next, the Pearson-POWA and Pearson-IPOWA are defined and added to the CRITIC methodology. Furthermore, the IPOWA CRITIC and the extensions IPOWA-OWA-S-CRITIC, IPOWA-OWA-W-CRITIC, and IPOWA-OWA-S-W-CRITIC are proposed to obtain the multicriteria score. The study is concluded with an empirical application, and the results are discussed to assess the applicability of this strategy. Finally, the main conclusions obtained are presented.

## 2 Materials and methods

### 2.1 Ordered weighted average

**Definition 1**. An ordered weighed average (OWA) operator (Yager, 1988) of dimension n is a mapping of $F_{\text{OWA}}:R^{n}\to R$ that has an associated weighting vector *W* = [ω_1_, ω_2_, …, ω_*n*_], such that ω_*i*_ ∈ [0, 1] and $\overset{n}{\underset{i=1}{\sum }}\omega_{i}=1$ defined as:


(1) $$\begin{array}{l} F_{OWA}\left(a_{1},a_{2},...,a_{n}\right)=\sum_{j=1}^{n}\omega_{j}b_{j} \end{array}$$

where *b*_*j*_ is the *j*^*th*^ largest of the *a*_*i*_.

The OWA operator is a non-linear function of elements, since it implies an ordering process. It presents the properties of commutativity, monotonicity, and boundedness:

- Commutativity: The initial ordering of the arguments does not matter.
- Monotonicity: $F_{OWA}\left(a_{1},a_{2},...,a_{n}\right)\geq F_{OWA}\left(a_{1}^{*},a_{2}^{*},...,a_{n}^{*}\right)$ if $a_{i}\geq a_{i}^{*}$ for all *i*.
- Boundedness: *Min*(*a*_1_, ..., *a*_*n*_) ≤ *F*_*OWA*_(*a*_1_, ..., *a*_*n*_) ≤ *Max*(*a*_1_, ..., *a*_*n*_).

An immediate application of boundness is idempotency: if *a*_*j*_ = *a* for all j, then *F*(*a*_*i*_, ..., *a*_*n*_) = *a*.

**Definition 2**. A Probabilistic OWA operator (POWA) of dimension n (Merigó, 2008, 2009) is a mapping of $F_{\text{POWA}}:R^{n}\to R$ with two associated weighting vectors *W* and *V* of dimension n, such that ω_*j*_ and *v*_*i*_ ∈ [0, 1] and $\overset{n}{\underset{j=1}{\sum }}\omega_{j}=1$ and $\overset{n}{\underset{i=1}{\sum }}v_{i}=1$ :


(2) $$\begin{array}{l} F_{POWA}\left(a_{1},...,a_{n}\right)=\sum_{j=1}^{n}{\hat{\upsilon }}_{j}b_{j} \end{array}$$

where *b*_*j*_ is the *j*^th^ largest of the *a*_*i*_, each argument *a*_*i*_ has an associated weight (probability) *v*_*i*_, ${\hat{\nu }}_{j}=\delta \omega_{j}+\left(1-\delta \right)\nu_{j}$ with δ ∈ [0, 1], and ν_*j*_ is the weight (probability), with ν_*i*_ ordered according to *b*_*j*_, that is, according to the *j*^th^ largest of the *a*_*i*_. If δ = 0 or ω_*j*_ = 1/*n* for all the *b*_*j*_, a probabilistic mean is obtained; on the contrary, if δ = 1 o *v*_*j*_ = 1/*n* for all the *b*_*j*_, the OWA operator is obtained.

The induced ordered weighted average operator (IOWA) introduced by Yager and Filev (1999) uses a second variable, the induced variable, to perform the ordering as a prior step to its aggregation.

Definition 3. An IOWA operator of dimension n is a mapping of $F_{\text{IOWA}}:R^{n}\times R^{n}\to R$; it has an associated weighting vector *W* of dimension n , such that ω_*i*_ ∈ [0, 1] and $\overset{n}{\underset{i=1}{\sum }}\omega_{i}=1$ :


(3) $$\begin{array}{l} F_{IOWA}\left(\langle u_{1},a_{1}\rangle ,\langle u_{2},a_{2}\rangle ,...,\langle u_{n},a_{n}\rangle \right)=\overset{n}{\underset{j=1}{\sum }}\omega_{j}b_{j} \end{array}$$

Where *b*_*j*_ is the *a*_*i*_ value of the IOWA pair 〈*u*_*i*_, *a*_*i*_〉 having the *j*^*th*^ largest *u*_*i*_, *u*_*i*_ is the order inducing variable, and *a*_*i*_ is the argument variable.

The induced probabilistic ordered weighted average (IPOWA) is an aggregation operator that uses probability and the OWA operator. Thus, the reordering of the values is performed according to the induced variable that represents a complex process of reordering of the individual distances formed by comparing two sets (Merigó and Casanovas, 2010). This contribution is interesting, as it combines the possibility of occurrence of certain results and the attitudinal character of decision-makers, who assess using inductive variables in order to represent their attitude completely. For this, it considers aspects such as the degree of optimism, psychological aspects, or the pressure of time.

**Definition 4**. An IPOWA operator (Merigó, 2014) of dimension n is a mapping of $F_{IPOWA}:R^{n}\times R^{n}\to R$ that has the associated weighting vectors *W* and of dimension n, such that ω_*j*_ and υ_*i*_ ∈ [0, 1] and $\sum_{j=1}^{n}\omega_{j}=1$ and $\sum_{i=1}^{n}\upsilon_{i}=1$:


(4) $$\begin{array}{l} F_{IPOWA}\left(\langle a_{1},u_{1}\rangle ,...,\langle a_{n},u_{n}\rangle \right)=\overset{n}{\underset{j=1}{\sum }}{\hat{\upsilon }}_{j}b_{j} \end{array}$$

Where *b*_*j*_ is the *a*_*i*_ value of the IOWA pair 〈*u*_*i*_, *a*_*i*_〉 having the *j*^th^ largest *u*_*i*_, *u*_*i*_ is the order inducing variable, each argument *a*_*i*_ is the argument variable with an associated weight (probability) *v*_*i*_, ${\hat{v}}_{j}=\delta \omega_{j}+\left(1-\delta \right)v_{j}$ with δ ∈ [0, 1], and *v*_*j*_ is the weight (probability), with *v*_*i*_ ordered according to *b*_*j*_, that is, according to the *j*^th^ largest of the *u*_*i*_.

### 2.2 Variance, covariance, and correlation coefficient

In this section, some previous concepts, such as the OWA-variance and the OWA-covariance, are analyzed and the Pearson-OWA operator is proposed. These elements will allow for the development of a new methodology named OWA-CRITIC, which allows the ordering of different alternatives, based on a multicriteria system, and the introduction of the possibility that Pearson's correlation coefficient is obtained based on a reordering of the elements to which weights are assigned, not the element itself, but to the position they occupy in the set.

**Definition 5**. Pearson's correlation coefficient measures the linear relationship between two variables *A* = {*a*_1_, …, *a*_*n*_} and *B* = {*b*_1_, …, *b*_*n*_} whose means are μ_*a*_ and μ_*b*_, respectively. Each argument (*a*_*i*_ − μ_*a*_)(*b*_*i*_ − μ_*b*_) has an associated weight *v*_*i*_ with $\overset{n}{\underset{i=1}{\sum }}v_{i}=1$ and *v*_*i*_ ∈ [0, 1], each argument ${\left(a_{i}-\mu_{a}\right)}^{2}$ has an associated weight $\nu_{i}^{a}$ with $\overset{n}{\underset{i=1}{\sum }}\nu_{i}^{a}=1$ and $\nu_{i}^{a}\in \left[0,1\right]$, each argument ${\left(b_{i}-\mu_{b}\right)}^{2}$ has an associated weight $v_{i}^{b}$ with $\overset{n}{\underset{i=1}{\sum }}v_{i}^{b}=1$ and $v_{i}^{b}\in \left[0,1\right]$, and can be defined as $F_{\text{Pearson}}\left(\langle a_{1},b_{1}\rangle ,\ldots ,\langle a_{n},b_{n}\rangle \right)=\frac{\overset{n}{\underset{i=1}{\sum }}v_{i}\left(a_{i}-\mu_{a}\right)\left(b_{i}-\mu_{b}\right)}{\sqrt{\overset{n}{\underset{i=1}{\sum }}v_{i}^{a}{\left(a_{i}-\mu_{a}\right)}^{2}}\sqrt{\overset{n}{\underset{i=1}{\sum }}v_{i}^{b}{\left(b_{i}-\mu_{b}\right)}^{2}}}$. For the case in which $v_{i}=v_{i}^{a}=v_{i}^{b}=1/n$ for all the *i*, we obtain


(5) $$\begin{array}{r} F_{Pearson}\left(\langle a_{1},b_{1}\rangle ,...,\langle a_{n},b_{n}\rangle \right)\\ =\frac{\sum_{i=1}^{n}\left(a_{i}-\mu_{a}\right)\left(b_{i}-\mu_{b}\right)}{\sqrt{\sum_{i=1}^{n}{\left(a_{i}-\mu_{a}\right)}^{2}}\sqrt{\sum_{i=1}^{n}{\left(b_{i}-\mu_{b}\right)}^{2}}} \end{array}$$

**Definition 6**. The variance OWA operator (Yager, 1996b) of dimension n is a mapping of $F_{Var-OWA}:R^{n}\to R$ that has an associated weighting vector *W* = [ω_1_, ω_2_, ..., ω_*n*_], such that ω_*i*_ ∈ [0, 1] and defined as


(6) $$\begin{array}{l} F_{Var-OWA}\left(a_{1},a_{2},...,a_{n}\right)=\sum_{j=1}^{n}\omega_{j}D_{j} \end{array}$$

where *D*_*j*_ is the *j*^*th*^ largest of the ${\left(a_{i}-\mu \right)}^{2}$, *a*_*i*_ is the argument variable, and μ is the average (in this case, the OWA operator).

The Var-OWA accomplishes properties similar to other OWA operators, including commutativity, monotonicity, and boundedness. When considering, it becomes the classical variance.

**Definition 7**. The covariance OWA operator (Merigó, 2011) of dimension n is a mapping of $F_{Covar-OWA}:R^{n}\times R^{n}\to R$ that has an associated weighting vector, such that and defined as


(7) $$\begin{array}{l} F_{Covar-OWA}\left(\langle a_{1},b_{1}\rangle ,...,\langle a_{n},b_{n}\rangle \right)=\sum_{j=1}^{n}\omega_{j}K_{j} \end{array}$$

where *K*_*j*_ is the *j*^th^ largest of the (*a*_*i*_ − μ_*a*_)(*b*_*i*_ − μ_*b*_), *a*_*i*_ is the argument variable of the first set of elements *A* = {*a*_1_, …, *a*_*n*_}, *b*_*i*_ is the argument variable of the second set of elements *B* = {*b*_1_, …, *b*_*n*_}, and μ_*a*_ and μ_*b*_ are the mean of the sets *A* and *B*, respectively.

The Covar-OWA accomplishes properties similar to other OWA operators, including commutativity, monotonicity, and boundedness. When considering ω_*i*_ = 1/*n*, it becomes the classical covariance.

The OWA operator can also be implemented into Pearson's correlation coefficient. The use of Pearson-OWA is proposed next, which allows modifying the process of aggregation of the squares of the differences with respect to the mean, and the products of the differences with respect to the means of the two variables to be compared, assigning a higher or lower importance as a function of the degree of optimism or pessimism of the decision-maker.

**Definition 8**. Pearson-OWA of dimension n is a mapping of $F_{Pearson-OWA}:R^{n}\times R^{n}\to R$ that has an associated weighting vector *W* = [ω_1_, ω_2_, ..., ω_*n*_], such that and $\sum_{i=1}^{n}\omega_{i}=1$ defined as:


(8) $$\begin{array}{r} F_{Pearson-OWA}\left(\langle a_{1},b_{1}\rangle ,...,\langle a_{n},b_{n}\rangle \right)\\ =\frac{\sum_{j=1}^{n}\omega_{j}\left(a_{j}-\mu_{a}\right)\left(b_{j}-\mu_{b}\right)}{\sqrt{\sum_{j=1}^{n}\omega_{j}{\left(a_{j}-\mu_{a}\right)}^{2}}\sqrt{\sum_{j=1}^{n}\omega_{j}{\left(b_{j}-\mu_{a}\right)}^{2}}} \end{array}$$

Where *a*_*j*_ and *b*_*j*_ are the *j*^*th*^ largest arguments in the sets of elements *A* = {*a*_1_, ..., *a*_*n*_} and *B* = {*b*_1_, ..., *b*_*n*_}, and μ_*a*_ and μ_*b*_ are the mean of the sets *A* and *B*, respectively. When considering ω_*i*_ = 1/*n*, it becomes the classical covariance.

The Pearson-OWA accomplishes the properties of the OWA operators: symmetry and boundedness, but not monotonicity.

Some special cases are as follows: if ω_*j*_ = 0, ∀j ≠ k y ω_*k*_ = 1, we obtain the maximum as an absolute value, that is, the maximum or the minimum, and if ω_*j*_ = 1/*n*, ∀j we have the traditional Pearson's coefficient.

The coefficient *F*_*Pearson*−*OWA*_ can be calculated in different ways depending on whether the OWA operator is considered (Table 1). For the study of the OWA variance and the OWA covariance, please see Yager (1996b, 2006). The study can be completed using different types of OWA, such as the maximum [ω = (1, 0, ..., 0)], the minimum [ω = (0, ..., 0, 1)], or the arithmetic mean [ω = (1/*n*, ..., 1/*n*)] where n is the number of elements of each variable, as well as weights that only depend on the values of the variables, or additive neat OWA ω = (ω_1_, …, ω_*n*_), where $\omega_{1}=f\left(x_{i}\right)/\sum_{i=1}^{n}f\left(x_{i}\right)$.

**Table 1:** Pearson-OWA analysis.

**Case**	**Expression *F*_*Pearson*−*OWA*_**	**Description**
1	$\frac{\sum_{j=1}^{n}\omega_{j}\left(a_{j}-\mu_{a}\right)\left(b_{j}-\mu_{b}\right)}{\sqrt{\sum_{j=1}^{n}\omega_{j}{\left(a_{j}-\mu_{a}\right)}^{2}}\sqrt{\sum_{j=1}^{n}\omega_{j}{\left(b_{j}-\mu_{b}\right)}^{2}}}$	The covariance is obtained with OWA means for the differences in variables a and b and the variances with OWA means for the differences in variables a and b
2	$\frac{\sum_{j=1}^{n}\omega_{j}\left(a_{j}-ā\right)\left(b_{j}-\mu_{b}\right)}{\sqrt{\sum_{j=1}^{n}\omega_{j}{\left(a_{j}-ā\right)}^{2}}\sqrt{\sum_{j=1}^{n}\omega_{j}{\left(b_{j}-\mu_{b}\right)}^{2}}}$	The covariance is obtained with normal means for the differences in variable a, and with OWA means for the differences in variable b. The variances are obtained with normal means for the differences in variable a, and OWA means for the differences in variable b.
3	$\frac{\sum_{j=1}^{n}\omega_{j}\left(a_{j}-\mu_{a}\right)\left(b_{j}-\bar{b}\right)}{\sqrt{\sum_{j=1}^{n}\omega_{j}{\left(a_{j}-\mu_{a}\right)}^{2}}\sqrt{\sum_{j=1}^{n}\omega_{j}{\left(b_{j}-\bar{b}\right)}^{2}}}$	The covariance is obtained with OWA means for the differences in variable a, and with normal means for the differences in variable b. The variances are obtained with OWA means for the differences in variable a, and normal means for the differences in variable b.
4	$\frac{\sum_{j=1}^{n}\omega_{j}\left(a_{j}-ā\right)\left(b_{j}-\bar{b}\right)}{\sqrt{\sum_{j=1}^{n}\omega_{j}{\left(a_{j}-\mu_{a}\right)}^{2}}\sqrt{\sum_{j=1}^{n}\omega_{j}{\left(b_{j}-\mu_{b}\right)}^{2}}}$	The covariance is obtained with normal means for the differences in variables a and b, and the variances are obtained with the OWA means for the differences in a and b.
5	$\frac{\sum_{j=1}^{n}\omega_{j}\left(a_{j}-\mu_{a}\right)\left(b_{j}-\mu_{b}\right)}{\sqrt{\sum_{j=1}^{n}\omega_{j}{\left(a_{j}-ā\right)}^{2}}\sqrt{\sum_{j=1}^{n}\omega_{j}{\left(b_{j}-\bar{b}\right)}^{2}}}$	The covariance is obtained with OWA means for the differences in a and b, and the variances are obtained with normal means for the differences in a and b.
6	$\frac{\sum_{j=1}^{n}\omega_{j}\left(a_{j}-ā\right)\left(b_{j}-\mu_{b}\right)}{\sqrt{\sum_{j=1}^{n}\omega_{j}{\left(a_{j}-\mu_{a}\right)}^{2}}\sqrt{\sum_{j=1}^{n}\omega_{j}{\left(b_{j}-\mu_{b}\right)}^{2}}}$	The covariance is obtained with normal means for the differences in a and with OWA means for the differences in b, and the variances are obtained with the OWA means for the differences in a and b.
7	$\frac{\sum_{j=1}^{n}\omega_{j}\left(a_{j}-\mu_{a}\right)\left(b_{j}-\bar{b}\right)}{\sqrt{\sum_{j=1}^{n}\omega_{j}{\left(a_{j}-\mu_{a}\right)}^{2}}\sqrt{\sum_{j=1}^{n}\omega_{j}{\left(b_{j}-\mu_{b}\right)}^{2}}}$	The covariance is obtained with OWA means for the differences in a and with normal means for the differences in b, and the variances are obtained with the OWA means for the differences in a and b.
8	$\frac{\sum_{j=1}^{n}\omega_{j}\left(a_{j}-ā\right)\left(b_{j}-\mu_{b}\right)}{\sqrt{\sum_{j=1}^{n}\omega_{j}{\left(a_{j}-ā\right)}^{2}}\sqrt{\sum_{j=1}^{n}\omega_{j}{\left(b_{j}-\bar{b}\right)}^{2}}}$	The covariance is obtained with normal means for the differences in a and with OWA means for the differences in b, and the variances are obtained with normal means for the differences in a and b.
9	$\frac{\sum_{j=1}^{n}\omega_{j}\left(a_{j}-\mu_{a}\right)\left(b_{j}-\bar{b}\right)}{\sqrt{\sum_{j=1}^{n}\omega_{j}{\left(a_{j}-ā\right)}^{2}}\sqrt{\sum_{j=1}^{n}\omega_{j}{\left(b_{j}-\bar{b}\right)}^{2}}}$	The covariance is obtained with OWA means for the differences in a and with normal means for the differences in b, and the variances are obtained with normal means for the differences in a and b.
10	$\frac{\sum_{j=1}^{n}\omega_{j}\left(a_{j}-ā\right)\left(b_{j}-\bar{b}\right)}{\sqrt{\sum_{j=1}^{n}\omega_{j}{\left(a_{j}-ā\right)}^{2}}\sqrt{\sum_{j=1}^{n}\omega_{j}{\left(b_{j}-\mu_{b}\right)}^{2}}}$	The covariance is obtained with normal means for the differences in a and b, and the variances are obtained with normal means for the differences in a and OWA means for the differences in b.
11	$\frac{\sum_{j=1}^{n}\omega_{j}\left(a_{j}-ā\right)\left(b_{j}-\bar{b}\right)}{\sqrt{\sum_{j=1}^{n}\omega_{j}{\left(a_{j}-\mu_{a}\right)}^{2}}\sqrt{\sum_{j=1}^{n}\omega_{j}{\left(b_{j}-\bar{b}\right)}^{2}}}$	The covariance is obtained with normal means for the differences in a and b, and the variances are obtained with the OWA means for the differences in a and normal means for the differences in b.
12	$\frac{\sum_{j=1}^{n}\omega_{j}\left(a_{j}-ā\right)\left(b_{j}-\mu_{b}\right)}{\sqrt{\sum_{j=1}^{n}\omega_{j}{\left(a_{j}-\mu_{a}\right)}^{2}}\sqrt{\sum_{j=1}^{n}\omega_{j}{\left(b_{j}-\bar{b}\right)}^{2}}}$	The covariance is obtained with normal means for the differences in a and OWA means for the differences in b, and the variances are obtained with OWA means for the differences in a and normal means for the differences in b.
13	$\frac{\sum_{j=1}^{n}\omega_{j}\left(a_{j}-\mu_{a}\right)\left(b_{j}-\bar{b}\right)}{\sqrt{\sum_{j=1}^{n}\omega_{j}{\left(a_{j}-ā\right)}^{2}}\sqrt{\sum_{j=1}^{n}\omega_{j}{\left(b_{j}-\mu_{b}\right)}^{2}}}$	The covariance is obtained with OWA means for the differences in a and normal means for the differences in b, and the variances are obtained with normal means for the differences in a and OWA means for the differences in b.
14	$\frac{\sum_{j=1}^{n}\omega_{j}\left(a_{j}-\mu_{a}\right)\left(b_{j}-\mu_{b}\right)}{\sqrt{\sum_{j=1}^{n}\omega_{j}{\left(a_{j}-ā\right)}^{2}}\sqrt{\sum_{j=1}^{n}\omega_{j}{\left(b_{j}-\mu_{b}\right)}^{2}}}$	The covariance is obtained with OWA means for the differences in a and b, and the variances are obtained with normal means for the differences in a and OWA means for the differences in b.
15	$\frac{\sum_{j=1}^{n}\omega_{j}\left(a_{j}-\mu_{a}\right)\left(b_{j}-\mu_{b}\right)}{\sqrt{\sum_{j=1}^{n}\omega_{j}{\left(a_{j}-\mu_{a}\right)}^{2}}\sqrt{\sum_{j=1}^{n}\omega_{j}{\left(b_{j}-\bar{b}\right)}^{2}}}$	The covariance is obtained with OWA means for the differences in a and b, and the variances are obtained with OWA means for the differences in a and normal means for the differences in b.
16	$\frac{\sum_{j=1}^{n}\omega_{j}\left(a_{j}-ā\right)\left(b_{j}-\bar{b}\right)}{\sqrt{\sum_{j=1}^{n}\omega_{j}{\left(a_{j}-ā\right)}^{2}}\sqrt{\sum_{j=1}^{n}\omega_{j}{\left(b_{j}-\bar{b}\right)}^{2}}}$	The covariance is obtained with normal means for the differences in a and b, and the variances are obtained with normal means for the differences in a and b.

On some occasions, aside from the attitudinal character of the decision-maker, which is introduced through the use of the OWA, objective information is available about the possibility of the occurrence of certain results, or the probability of application of the results, so that they will have to be used. Pearson-POWA is proposed, as it allows combining both types of information.

**Definition 9**. The Pearson-POWA of dimension n is a mapping of $F_{Pearson-POWA}:R^{n}\times R^{n}\to R$ that has an associated weighting vector *W* of dimension n, such that ω_*i*_ and $\sum_{i=1}^{n}\omega_{i}=1$:


(9) $$\begin{array}{r} F_{Pearson-POWA}\left(\langle a_{1},b_{1}\rangle ,...,\langle a_{n},b_{n}\rangle \right)\\ =\frac{\sum_{i=1}^{n}{\hat{\upsilon }}_{i}\left(a_{j}-\mu_{a}\right)\left(b_{j}-\mu_{b}\right)}{\sqrt{\sum_{i=1}^{n}{\hat{\upsilon }}_{i}{\left(a_{j}-\mu_{a}\right)}^{2}}\sqrt{\sum_{i=1}^{n}{\hat{\upsilon }}_{i}{\left(b_{j}-\mu_{a}\right)}^{2}}} \end{array}$$

Where *a*_*j*_ and *b*_*j*_ are the *j*^th^ largest arguments in the sets of elements *A* = {*a*_1_, …, *a*_*n*_} and *B* = {*b*_1_, …, *b*_*n*_}, and μ_*a*_ and μ_*b*_ are the mean of the sets *A* and *B*, respectively. The arguments $\left(a_{j}-\mu_{a}\right)\left(b_{j}-\mu_{b}\right),{\left(a_{j}-\mu_{a}\right)}^{2}$, and ${\left(b_{j}-\mu_{a}\right)}^{2}$ have an associated weight (probability) *v*_*i*_ ordered according to $\left(a_{j}-\mu_{a}\right)\left(b_{j}-\mu_{b}\right),{\left(a_{j}-\mu_{a}\right)}^{2}$, and ${\left(b_{j}-\mu_{a}\right)}^{2}$, respectively, ${\hat{\nu }}_{j}=\delta \omega_{j}+\left(1-\delta \right)v_{j}$ with δ ∈ [0, 1]. If δ = 0 or ω_*j*_ = 1/*n* for all the $\left(a_{j}-\mu_{a}\right)\left(b_{j}-\mu_{b}\right),{\left(a_{j}-\mu_{a}\right)}^{2}$ and ${\left(b_{j}-\mu_{a}\right)}^{2}$, the Pearson probability is obtained, in which the weight of each sum is given by the assigned probabilities, while if δ = 1 or *v*_*j*_ = 1/*n* for all the $\left(a_{j}-\mu_{a}\right)\left(b_{j}-\mu_{b}\right),{\left(a_{j}-\mu_{a}\right)}^{2}$, and ${\left(b_{j}-\mu_{a}\right)}^{2}$, the *F*_Pearson-OWA_ operator is obtained.

Pearson-IPOWA combines the concept of the probability of occurrence or applicability of specific events with the ordering of elements according to the attitudinal character of the decision maker, but in this case, this ordering is performed based on inducting variables of order, which can represent a broad range of aspects, such as the degree of optimism or pessimism, or psychological or time pressure aspects.

**Definition 10**. The Pearson-IPOWA of dimension n is a mapping of $F_{\text{Pearson-IPOWA}}:R^{n}\times R^{n}\times R^{n}\to R$ that has an associated weighting vector *W* of dimension n, such that ω_*i*_ ∈ [0, 1] and $\overset{n}{\underset{i=1}{\sum }}\omega_{i}=1$, defined as:


(10) $$\begin{array}{r} F_{Pearson-IPOWA}\left(\left[\langle a_{1},b_{1}\rangle ,u_{1}\right],...,\left[\langle a_{n},b_{n}\rangle ,u_{n}\right]\right)\\ =\frac{\sum_{j=1}^{n}{\hat{\upsilon }}_{j}D_{j}}{\sqrt{\sum_{j=1}^{n}{\hat{\upsilon }}_{j}K_{j}}\sqrt{\sum_{j=1}^{n}{\hat{\upsilon }}_{i}H_{j}}} \end{array}$$

Where *D*_*j*_, *K*_*j*_, *H*_*j*_ are the values of $\left(a_{j}-\mu_{a}\right)\left(b_{j}-\mu_{b}\right),{\left(a_{j}-\mu_{a}\right)}^{2}$, and ${\left(b_{j}-\mu_{a}\right)}^{2}-$ respectively, having the *j*^th^ largest *u*_*i*_, *u*_*i*_ is the order inducing variable. Each argument, *D*_*i*_, *K*_*i*_, and *H*_*i*_, has an associated weight (probability) *v*_*i*_ ordered according to the largest of the $u_{i},{\hat{v}}_{j}=\delta \omega_{j}+\left(1-\delta \right)v_{j}$ with δ ∈ [0, 1], and *v*_*j*_ is the weight (probability); *v*_*i*_ is ordered according to *D*_*j*_, that is, according to the *j* th largest of the *u*_*i*_. If δ = 0 o ω_*j*_ = 1/*n* for all the *D*_*j*_, *K*_*j*_, *H*_*j*_, the Pearson probability *F*_Pearson-POWA_ is obtained, on the other hand, if δ = 1 or *v*_*j*_ = 1/*n* for all the *D*_*j*_, *K*_*j*_, *H*_*j*_ the induced Pearson-OWA, *F*_Pearson-IOWA_ is obtained.

### 2.3 IPOWA-CRITIC and its extensions

The following section proposes diverse extensions of the CRITIC methodology, using the operator proposed in the previous section, to obtain the correlation between the values of the different criteria and the use of OWA to obtain the multicriteria score.

**Figure 1:**
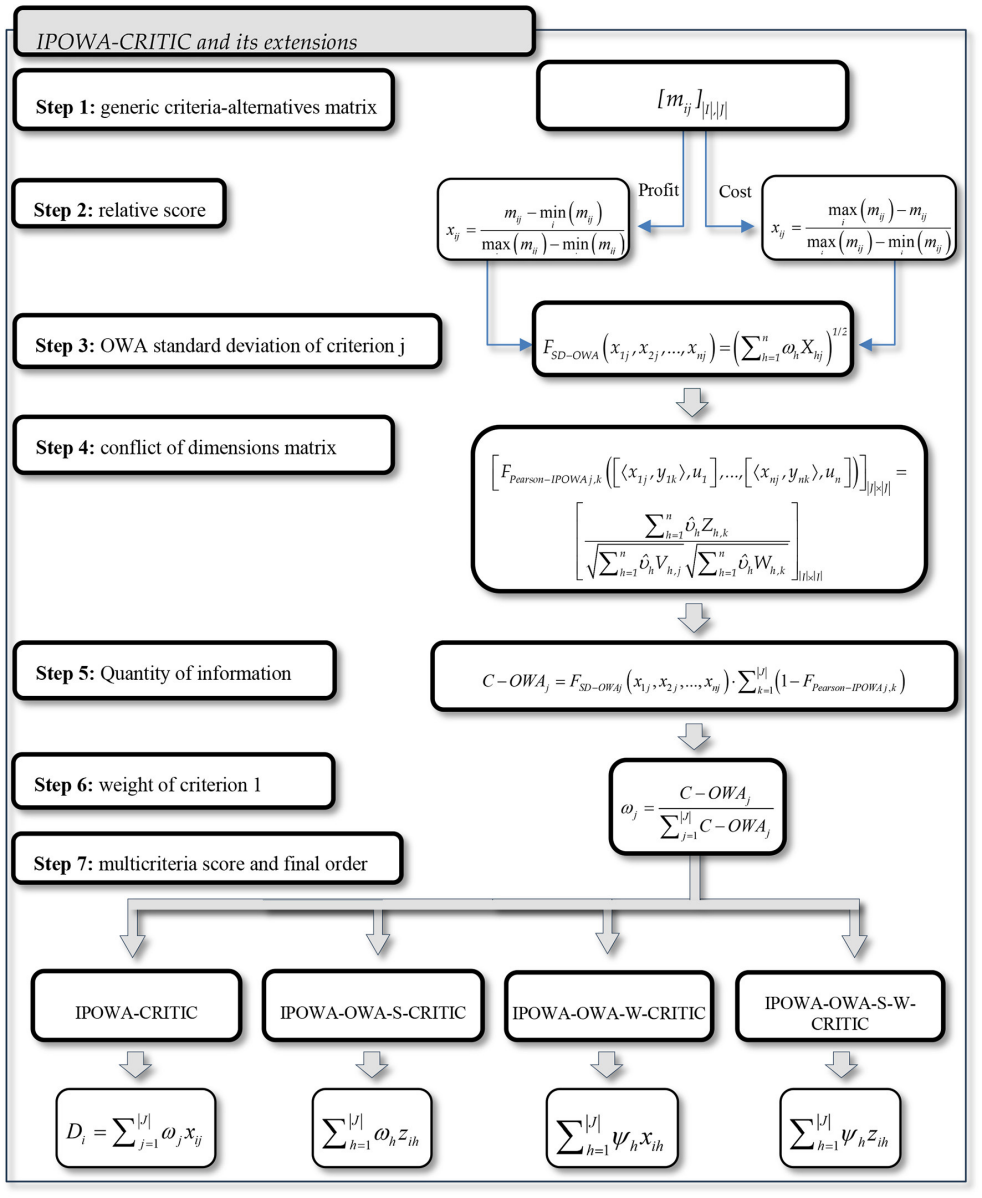
IPOWA-CRITIC and its extensions process.

The CRITIC methodology uses the multicriteria system to order a set of alternatives. This study is an extension of the proposal by Diakoulaki et al. (1995). The objective is to order a set of alternatives *A* = {1, ..., |*I*|}, with cardinality |*I*|, and according to a set of criteria *C* = {1, ..., |*J*|} with cardinality |*J*|. The following steps are proposed (Figure 1):

Step 1. The generic criteria-alternatives matrix shows the *m*_*ij*_ values of alternative *i* for criteria *j* (Table 2).

**Table 2:** Values of criteria used (C_1_, …, C_j_) to rank the treatments applied (A_1_,…, A_i_).

	**Criteria (** ***j*** **)**			
**Alternatives (i)**	**C**_1_	**C**_2_	**…**	**C**_|J|_
A_1_				
A_2_				
…			m_ij_	
A_|I|_				

Step 2. Transformation of the generic criteria-alternatives matrix into the relative score or closeness matrix to the ideal values [*x*_*ij*_]. For each *j* criterion, the minimum is obtained, $\underset{i}{\min}\left(m_{ij}\right)$, as well as the maximum $\underset{i}{\max}\left(m_{ij}\right)$ of all the alternatives, in a similar manner to the crisp case. When the ideal value is the maximum value (benefits), the closeness to the ideal value is obtained as:


(11) $$\begin{array}{l} x_{ij}=\frac{m_{ij}-\underset{i}{\min}\left(m_{ij}\right)}{\underset{i}{\max}\left(m_{ij}\right)-\underset{i}{\min}\left(m_{ij}\right)} \end{array}$$

and the ideal value is the minimum value (cost):


(12) $$\begin{array}{l} x_{ij}=\frac{\underset{i}{\max}\left(m_{ij}\right)-m_{ij}}{\underset{i}{\max}\left(m_{ij}\right)-\underset{i}{\min}\left(m_{ij}\right)} \end{array}$$

Step 3. Obtaining the OWA standard deviation of criterion j, for each vector *x*_*j*_ = (*x*_1*j*_, *x*_2*j*_, ..., *x*_|*I*|*j*_) of the transformed matrix, from Equation 6


(13) $$\begin{array}{l} F_{SD-OWA}\left(x_{1j},x_{2j},...,x_{nj}\right)={\left(\sum_{h=1}^{n}\omega_{h}X_{hj}\right)}^{1/2} \end{array}$$

Where *X*_*hj*_ is the *h*^*th*^ largest value of ${\left(x_{ij}-\mu_{j}\right)}^{2},\omega_{i}\in \left[0,1\right]$, and $\overset{n}{\underset{i=1}{\sum }}\omega_{i}=1$.

Step 4. Construction of the conflict of dimensions matrix |*J*| × |*J*|, whose generic term *F*_Pearson-IPOWA j, k_ represents the correlation between elements *x*_*j*_ and *x*_*k*_. Therefore, the weaker the relationship between the criteria *j* and *k*, the smallest the value of *F*_Pearson-IPOWA*j, k*_ will be.


(14) $$\begin{array}{r} {F_{Pearson-IPOWA}}_{j,k}\left(\left[\langle x_{1j},y_{1k}\rangle ,u_{1}\right],...,\left[\langle x_{nj},y_{nk}\rangle ,u_{n}\right]\right)\\ =\frac{\sum_{h=1}^{n}{\hat{\upsilon }}_{h}Z_{h,k}}{\sqrt{\sum_{h=1}^{n}{\hat{\upsilon }}_{h}V_{h,j}}\sqrt{\sum_{h=1}^{n}{\hat{\upsilon }}_{h}W_{h,k}}} \end{array}$$

where *Z*_*h, k*_, *V*_*h, j*_, and *W*_*h, k*_ are the values of $\left(x_{ij}-\mu_{xj}\right)\left(y_{ik}-\mu_{yk}\right),{\left(x_{ij}-\mu_{xj}\right)}^{2}$, and ${\left(x_{ik}-\mu_{xk}\right)}^{2}$, respectively, having the *h*^*th*^ largest *u*_*i*_, with *u*_*i*_ being the order inducing variable, and with μ_*xj*_ being the mean of the first set of values for criterion *j, X*_*j*_ = {*x*_1*j*_, …, *x*_*nj*_}, and μ_*y, k*_ the mean of the second set, *X*_*k*_ = {*x*_1*k*_, …, *x*_*nk*_}. Each *Z*_*i, k*_, *V*_*i, j*_, and *W*_*i, k*_ has an associated weight (probability) *v*_*i*_ with $\overset{n}{\underset{i=1}{\sum }}v_{i}$ and $v_{i}\in \left[0,1\right],{\hat{v}}_{h}=\delta \omega_{h}+\left(1-\delta \right)v_{h}$ with δ ∈ [0, 1] and *v*_*h*_ is the weight (probability), with *v*_*i*_ ordered according to the *h*^th^ largest *u*_*i*_, with $u_{i}=\overset{\left|J\right|}{\underset{j=1}{\sum }}x_{ij}$, that is, the sum of all the relative scores of the alternative *A*_*i*_. This probability represents the probability of occurrence, or in this particular case, the probability of commercially implementing this treatment. This coefficient will be obtained from the opinions of the experts related with the present project.

Step 5. Quantity of information *C* − *OWA*_*j*_ is emitted by the *j*^*th*^ criterion, which represents the quantity of information transmitted by said criterion, so that the more information transmitted, the greater the value of *C* − *OWA*_*j*_:


(15) $$\begin{array}{r} C-OWA_{j}=F_{SD-OWAj}\left(x_{1j},x_{2j},...,x_{nj}\right)\\ \cdot \sum_{k=1}^{\left|J\right|}\left(1-{F_{Pearson-IPOWA}}_{j,k}\right) \end{array}$$

Step 6. The weight of criterion *j* is obtained by normalizing the values of *C* − *OWA*_*j*_.


(16) $$\begin{array}{l} \omega_{j}=\frac{C-OWA_{j}}{\sum_{j=1}^{\left|J\right|}C-OWA_{j}} \end{array}$$

Step 7. Obtaining the multicriteria score for alternative *i*: For this step, four alternatives are proposed depending on how the OWA is used for the relative scores, the weights, for both, or none of them.

7.1. Multicriteria score with IPOWA-CRITIC to order the different alternatives. The Multicriteria score with IPOWA-CRITIC (*D*_*i*_) is obtained as the product of the weights obtained in Equation 16 by the relative scores obtained in Equations 11, 12.


(17) $$\begin{array}{l} D_{i}=\sum_{j=1}^{\left|J\right|}\omega_{j}x_{ij} \end{array}$$

7.2. Multicriteria score with the IPOWA-OWA-S-CRITIC to order the different alternatives. The IPOWA-OWA-S-CRITIC multicriteria score for alternative *i* is a mapping of $F_{\text{IPOWA-OWA-S-CRIIC}}:R^{n}\to R$ that has the weighted vector obtained in Equation 16 associated: *W* = [ω_1_, ω_2_, …, ω_1|*J*|_] where ω_*i*_ ∈ [0, 1] and $\overset{n}{\underset{i=1}{\sum }}\omega_{i}=1$ is defined as:


(18) $$\begin{array}{l} F_{IPOWA-OWA-S-CRITIC}\left(x_{i1},x_{i2},...,x_{i|J|}\right)=\sum_{h=1}^{\left|J\right|}\omega_{h}z_{ih} \end{array}$$

where *z*_*ih*_ is the *h*^*th*^ largest of the *x*_*ij*_ (alternative *A*_*i*_), and ω_*h*_ is the weight obtained in Equation 16.

7.3. Multicriteria score with IPOWA-OWA-W-CRITIC to order the different alternatives. The IPOWA-OWA-S-CRITIC multicriteria score for alternative *i* is a mapping of $F_{IPOWA-OWA-W-CRITIC}:R^{n}\to R$ that has the weighted vector obtained in Equation 16 associated: *W* = [ω_1_, ω_2_, ..., ω_|*J*|_], where and $\sum_{i=1}^{n}\omega_{i}=1$ is defined as:


(19) $$\begin{array}{l} F_{IPOWA-OWA-W-CRITIC}\left(x_{i1},x_{i2},...,x_{i|J|}\right)=\sum_{h=1}^{\left|J\right|}\psi_{h}x_{ih} \end{array}$$

where ψ_*h*_ is the *h*^*th*^ largest of the ω_*j*_, and *x*_*ih*_ is the relative score obtained in Equations 11, 12.

7.4. Multicriteria score with IPOWA-OWA-S-W-CRITIC to order the different alternatives. The IPOWA-OWA-S-CRITIC multicriteria score for alternative *i* is a mapping of $F_{IPOWA-OWA-S-W-CRITIC}:R^{n}\to R$ that has the weighted vector obtained in Equation 16 associated: *W* = [ω_1_, ω_2_, ..., ω_|*J*|_], where ω_*i*_ ∈ [0, 1] and $\sum_{i=1}^{n}\omega_{i}=1$ is defined as:


(20) $$\begin{array}{l} F_{IPOWA-OWA-S-W-CRITIC}\left(x_{i1},x_{i2},...,x_{i|J|}\right)=\sum_{h=1}^{\left|J\right|}\psi_{h}z_{ih} \end{array}$$

where ψ_*h*_ is the *h*^*th*^ largest of the ω_*j*_ and *z*_*ih*_ is the *h*^*th*^ largest of the (alternative *A*_*i*_) obtained in Equations 11, 12.

### 2.4 Empirical application

Muchamiel tomatoes were grown in a multi-span mesh greenhouse (windbreak greenhouse) that was 26 m wide, 36 m long, and 4 m high until the gutter and 5 m to the ridge, located at the CIAGRO-UMH (Orihuela, Alicante, Spain, Latitude: 38° 05' 05” North; Longitude: 0° 56' 38” West). A short spring-summer cycle was used for 2 consecutive years. The first with a transplantation on 6^th^ March 2023 and harvesting of plants on 28^th^ June 2023. The second is between 4^th^ March and 28^th^ June 2024. Inside the greenhouse, three plots with different shading systems were set up: (i) without shade (W), (ii) with a fixed and conventional shade nets with 50% of reflection of the solar radiation (F), and (iii) a mobile mesh with photovoltaic shading (P). In each of the plots and on the exterior of the greenhouse, the mean values of the main climate variables were recorded at 10-min intervals, such as ambient temperature and humidity, as well as the intensity of the solar radiation. The mean values of the energy variables were also determined, related to the photovoltaic mesh, at 10-min intervals. In each plot, grafted plants (G) and non-grafted plants (N) were used, with two watering events, according to the needs of the crop, through Allen et al. (2006) method (complete irrigation, C), and with deficit irrigation at 60% of said value (D). The total number of plants used in each treatment was 36 plants. Agroecological strategies and techniques were followed during the management of the crop, such as the application of biostimulants and integrated pest management. Table 3 shows the treatments applied during the assay.

**Table 3:** Treatments (W, without shading; C, conventional shading; SF, photovoltaic shading; G, grafted plants; N, non-grafted plants; C, complete irrigation; and D, deficit irrigation).

**Key**	**Treatment**
A_1_	WGC
A_2_	WGD
A_3_	WNC
A_4_	WND
A_5_	FGC
A_6_	FGD
A_7_	FNC
A_8_	FND
A_9_	PGC
A_10_	PGD
A_11_	PNC
A_12_	PND

To establish the sustainability of the production strategies used, criteria related to the agronomical, physiological, and biochemical responses of the plant were used, such as production (kg ha^−1^) and quality, determined starting with the maturity index (°Brix/Acidity) and nutritional composition (%). The profit obtained (€ ha^−1^) with respect to the economic sustainability of the treatments was also considered. The water consumption (m^3^ ha^−1^) and CO_2_ fixation (t ha^−1^) allowed us to consider the environmental sustainability of the treatments. In addition, the social effect was determined starting from the labor used in each treatment. Table 4 shows the criteria used.

**Table 4:** Criteria taken into account to grade the treatments.

**Key**	**Criteria**
BI_1_	Production (kg ha^−1^)
BI_2_	Profit (€ ha^−1^)
BI_3_	Water consumption (m^3^ ha^−1^)
BI_4_	CO_2_ fixation (t ha^−1^)
BI_5_	Labor (€ ha^−1^)
BI_6_	Maturity index (°Brix/Acidity)
BI_7_	Nutritional composition (%)

Five experts on the subject were consulted to establish confidence in the results obtained in each of the criteria for each of the treatments utilized, and to support the making of decisions on its possible application at a commercial exploitation scale.

To improve the analysis of the sustainability of the agronomic strategies utilized, a sustainability index calculated from the IPOWA CRITIC assignment is proposed. The value of this index varies between 0 and 1. A value of 0 corresponds to the treatment with the worst results according to the criteria used (Table 4), and a value of 1 indicates the best treatment.

### 2.5 Statistical analysis

The results were statistically evaluated using an analysis of variance, ANOVA, with a 95% confidence interval. The differences between the means of the treatments were analyzed using the least significant difference test of Fisher (LSD) at a probability level of 95%. Significance levels were expressed as: ^*^ *p* < 0.05; ^**^ *p* < 0.01; ^***^ *p* < 0.001; NS not significant. The results of all treatments were analyzed in each of the 2 years of cultivation, and no significant differences were found between them. The results for the 1^st^ year of cultivation are presented here.

## 3 Results and discussion

The values of the IPOWA-CRITIC and its extensions were obtained according to the procedure described above.

Step 1. Identify the results matrix for each criterion and alternative (Table 5). The results obtained in each of the criteria by the treatments applied are shown in Table 5. As shown, treatment A_1_ (WGC) showed a production of 72,870 kg ha^−1^, higher than the rest. With respect to the water consumption, the treatments were mainly distributed into two groups, the ones that were irrigated at 100% of their needs (C), with a consumption of approximately 3,300 m^3^ ha^−1^, and those that were irrigated at 60% (D), with a consumption of approximately 2,000 m^3^ ha^−1^. As for CO_2_ fixation, this was higher in the treatments with photovoltaic shades due to the elimination of CO_2_ during the generation of the electrical energy consumed. The use of labor is related to production, being higher in the treatments with a higher production, such as WGC. The plants without shading had a higher maturity index value, followed by plants with fixed and conventional shading (F) and plants with photovoltaic shading. On its part, the nutritional composition of the tomatoes in the plot without shading was similar to that from the plot with photovoltaic shading (P), and both were inferior to that determined in the plot with F treatments.

**Table 5:** Values of the criteria used (from BI_1_ to BI_7_) to rank the treatments applied (from A_1_ to A_12_).

**Alternatives**	**BI_1_**	**BI_2_**	**BI_3_**	**BI_4_**	**BI_5_**	**BI_6_**	**BI_7_**
A_1_	72,870.00	69,588.63	3,336.17	−7,119.23	42,065.15	13.64	9.43
A_2_	67,450.00	61,757.88	1,963.68	−6,664.80	40,981.15	13.55	9.33
A_3_	58,400.00	48,917.63	3,311.43	−6,537.39	39,171.15	12.70	9.18
A_4_	56,800.00	47,962.88	1,999.19	−6,318.86	38,851.15	13.48	9.20
A_5_	37,000.00	5,022.63	3,321.91	−3,530.01	34,891.15	12.22	9.73
A_6_	37,600.00	8,027.88	2,013.90	−3,888.43	35,011.15	11.78	9.46
A_7_	40,400.00	16,517.63	3,315.13	−4,607.23	35,571.15	12.17	9.32
A_8_	32,200.00	3,682.88	1,975.35	−3,970.31	33,931.15	12.88	9.12
A_9_	44,600.00	38,249.43	3,294.27	9,929.74	38,314.35	11.33	9.53
A_10_	37,800.00	10,823.57	2,008.33	10,977.96	36,954.35	10.46	9.42
A_11_	48,600.00	33,713.32	3,258.37	9,118.41	39,114.35	11.46	9.33
A_12_	43,400.00	26,278.57	1,984.01	9,638.28	38,074.35	12.00	8.97

Step 2. To be able to compare all the criteria, they are standardized. The normalized values are shown in Table 6. Except for the consumption of water, all the criteria show profits, as it is better if the values are higher. In the case of water consumption, BI_3_, the value represents a cost, so it is better if this value is lower. Therefore, expression (11) was used for all, except for BI_3_, in which case, expression (12) was used. As can be observed, treatment A_1_ (WGC) has higher values in most of the criteria, except for water consumption and CO_2_ fixation, which show a null value, and nutritional composition (% nutrients), with a value of 0.605. Table 6 also shows the sum of the distances relative to the ideal values of each treatment, which will be used as an induced value in the case of using the IOWA. Moreover, the OWA standard deviation is shown, in agreement with the expression (Equation 13).

**Table 6:** Normalized values of the criteria used (from BI_1_ to BI_7_) to rank the treatments performed (from A_1_ to A_12_), sum of the relative distances to the ideal values, and values of the OWA standard deviation.

**Alternatives**	**BI_1_**	**BI_2_**	**BI_3_**	**BI_4_**	**BI_5_**	**BI_6_**	**BI_7_**	**Sum**
A_1_	1.000	1.000	0.000	0.000	1.000	1.000	0.605	4.605
A_2_	0.867	0.881	1.000	0.025	0.867	0.973	0.471	5.083
A_3_	0.644	0.686	0.018	0.032	0.644	0.706	0.276	3.007
A_4_	0.605	0.672	0.974	0.044	0.605	0.951	0.302	4.153
A_5_	0.118	0.020	0.010	0.198	0.118	0.552	1.000	2.018
A_6_	0.133	0.066	0.963	0.179	0.133	0.415	0.644	2.532
A_7_	0.202	0.195	0.015	0.139	0.202	0.538	0.460	1.750
A_8_	0.000	0.000	0.991	0.174	0.000	0.762	0.197	2.125
A_9_	0.305	0.524	0.031	0.942	0.539	0.271	0.732	3.345
A_10_	0.138	0.108	0.967	1.000	0.372	0.000	0.596	3.181
A_11_	0.403	0.456	0.057	0.897	0.637	0.312	0.473	3.236
A_12_	0.275	0.343	0.985	0.926	0.509	0.484	0.000	3.522
Mean	0.391	0.413	0.501	0.380	0.469	0.580	0.480	3.213

Step 3. Since the weights of each criterion in OWA-CRITIC depend on its dispersion, the OWA standard deviation of each criterion is obtained. As a prior step before obtaining the standard deviation of each criterion, Table 7 (column 2) shows the confidence of the results of the treatment for their subsequent passage to commercial exploitation, where 0 represents no confidence, and 1 represents maximum confidence. For this, five experts were consulted, and mean values were calculated. The third column in Table 7 shows the probability of each treatment, dividing the confidence of each treatment by the total sum of the treatments. The fifth column provides the weight ω_*i*_ assigned in expression (Equation 14) corresponding to , so that a higher weight is assigned to the totals that correspond to a treatment closer to ideal values. *F*_*Pearson*−*IPAOWA*_ uses the same weights but assigns a greater weight to the treatment with lower values in Table 6 (column 9). OWA standard deviation values of criterion *j* are presented in Table 8 (row 9).

**Table 7:** Level of confidence in the treatments and probability, and weight coefficients of for each position.

**Treatment**	**Confidence**	**Probability**	**Order**	**ω_*i*_ in *F*_*Pearson* − *IPOWA*_**
A_1_	0.200	0.057	1	0.200
A_2_	0.300	0.038	2	0.180
A_3_	0.300	0.057	3	0.170
A_4_	0.300	0.189	4	0.150
A_5_	0.100	0.151	5	0.130
A_6_	0.400	0.132	6	0.100
A_7_	0.500	0.094	7	0.020
A_8_	0.200	0.057	8	0.010
A_9_	0.800	0.075	9	0.010
A_10_	0.500	0.038	10	0.010
A_11_	0.700	0.019	11	0.010
A_12_	1.000	0.094	12	0.010
	Sum	1.000		1.000

**Table 8:** Pearson-IPOWA matrix and aggregation of values.

**Criteria**	**BI_1_**	**BI_2_**	**BI_3_**	**BI_4_**	**BI_5_**	**BI_6_**	**BI_7_**
BI_1_	1.000	0.967	−0.048	−0.578	0.925	0.807	0.080
BI_2_	0.967	1.000	−0.106	−0.427	0.956	0.721	0.083
BI_3_	−0.048	−0.106	1.000	−0.095	−0.099	0.226	−0.600
BI_4_	−0.578	−0.427	−0.095	1.000	−0.225	−0.847	−0.158
BI_5_	0.925	0.956	−0.099	−0.225	1.000	0.574	0.011
BI_6_	0.807	0.721	0.226	−0.847	0.574	1.000	−0.145
BI_7_	0.080	0.083	−0.600	−0.158	0.011	−0.145	1.000

Step 4. To analyze the conflict between criteria, Table 8 shows the Pearson-IPOWA considering δ = 0.6, that is, considering the weighting of its differences and quadratic differences with 60% of the OWA element and 40% of the probability element.

Step 5. Determination of the quantity of information emitted by the *j*^*th*^ criterion (Table 9, row 4) was obtained as the product of the standard deviation (Table 9, row 2) by the aggregates $\sum_{k=1}^{\left|J\right|}\left(1-{F_{Pearson-IPOWA}}_{j,k}\right)$ (Table 9, row 3).

**Table 9:** Standard deviation, aggregation of the Pearson-IPOWA, quantity of information emitted by the *j*^*th*^ criterion, and final weight of the criterion j.

**Criterion**	**BI_1_**	**BI_2_**	**BI_3_**	**BI_4_**	**BI_5_**	**BI_6_**	**BI_7_**
OWA SD	0.317	0.322	0.479	0.448	0.280	0.320	0.247
$\overset{\left|J\right|}{\underset{k=1}{\sum }}$(1−*F*_Pearson-IPOWA*j, k*_)	3.846	3.805	6.721	8.329	3.858	4.664	6.730
*C*−*OWA*_*j*_	1.217	1.226	3.223	3.734	1.082	1.494	1.662
ω_*j*_	0.089	0.090	0.236	0.274	0.079	0.110	0.122

Step 6. Obtaining the weight of the j^*th*^ criterion. The quotient of the amount of information emitted by criterion *j* divided by the sum of the amount of information emitted by all the criteria allows us to obtain the weight of criterion *j* (Table 9, row 5).

Step 7. Calculation of the multicriteria score.

7.1. Multicriteria score with IPOWA-CRITIC of alternative *i* (Table 10, column 2) is obtained by multiplying the weighting vectors from Table 9 (row 5) by the relative scores of Table 6 (row *A*_*i*_).

**Table 10:** Multicriteria score for the IPOWA CRITIC and its extensions.

**Treatment**	**IPOWA CRITIC**	**IPOWA-S- CRITIC**	**IPOWA-W- CRITIC**	**IPOWA-S-W- CRITIC**
A_1_	0.442	0.789	0.415	0.796
A_2_	0.632	0.789	0.668	0.828
A_3_	0.294	0.515	0.284	0.534
A_4_	0.546	0.619	0.588	0.724
A_5_	0.261	0.248	0.210	0.455
A_6_	0.429	0.350	0.449	0.515
A_7_	0.208	0.237	0.186	0.334
A_8_	0.389	0.271	0.435	0.495
A_9_	0.501	0.509	0.457	0.608
A_10_	0.627	0.481	0.639	0.638
A_11_	0.478	0.472	0.449	0.572
A_12_	0.635	0.513	0.681	0.659

7.2. Multicriteria score with IPOWA-S-CRITIC. The multicriteria score of the alternative *i* (Table 10, column 3) is obtained by multiplying the weighting vectors from Table 9 (row 5) by the relative scores *z*_*ih*_, *h* = 1, ...7, where *z*_*ih*_ is the highest *h*^*th*^ of the (Table 6) of the row corresponding to the alternative *A*_*i*_, *i* = 1, ..., 12.

Step 7.3. Multicriteria score with IPOWA-W-CRITIC. The multicriteria score of the alternative *i* (Table 10, column 4) is obtained by multiplying the weighting vector ψ_*h*_, *h* = 1, ..., 7, where ψ_*h*_ is the highest *h*^*th*^ of the ω_*j*_ from Table 9 (row 5) by the relative scores of Table 6 (row *A*_*i*_).

Step 7.4. Multicriteria score with IPOWA-S-W-CRITIC. The multicriteria score of the alternative *i* (Table 10, column 5) is obtained by multiplying the weighting vectors ψ_*h*_, *h* = 1, ..., 7, where ψ_*h*_ is the highest *h*^*th*^ of the from Table 9 (row 5) by the relative score *z*_*ih*_, *h* = 1, ...7, where *z*_*ih*_ is the highest *h*^*th*^ of the (Table 6, row *A*_*i*_) of the row corresponding to the alternative *A*_*i*_, *i* = 1, ..., 12.

### 3.1 Comparative analysis

In order to validate the proposed model, we proceeded to compare the results obtained by applying IPOWA-CRITIC, aside from IPOWA-S-CRITIC, IPOWA-W-CRITIC, and IPOWA-S-W-CRITIC, with other methodologies such as CRITIC, and other selected due to their simplicity, rationality, comprehensibility, good computational efficiency and ability to measure the relative performance for each alternative in a simple mathematical form, such as the scoring methods: Simple additive weighting, SAW (Podvezko, 2011), and Complex Proportional Assessment, COPRAS, based on the evaluation of different alternative through basic arithmetic operations, adding each normalized value of each criterion by its corresponding weight; and distance-based methods: Multicriteria optimization and compromise solution, VIKOR (Opricovic and Tzeng, 2004), and Technique for order of preference by similarity to ideal solution (TOPSIS), based on the calculation of each distance between each alternative and specific point.

Table 11 shows the rank of different alternatives. For CRITIC, TOPSIS, VIKOR, and SAW, the following was used as the weighting vector: ω = {0.20, 0.18, 0.17, 0.15, 0.13, 0.10, 0.07}, using a coefficient of 0.90 for the Manhattan distance and 0.10 for infinite one in the VIKOR method. As shown, the proposed extensions (IPOWA-S-CRITIC, IPOWA-W-CRITIC, IPOWA-S-W-CRITIC) show a very high correlation, obtained from Spearman's correlation coefficient (*r*_*s*_), with respect to the IPOWA CRITIC method, being higher than 0.64 in all cases, and also of the IPOWA CRITIC with respect to other methodologies such as CRITIC, TOPSIS, VIKOR and SAW, being higher than 0.76 in all of these cases.

**Figure 2:**
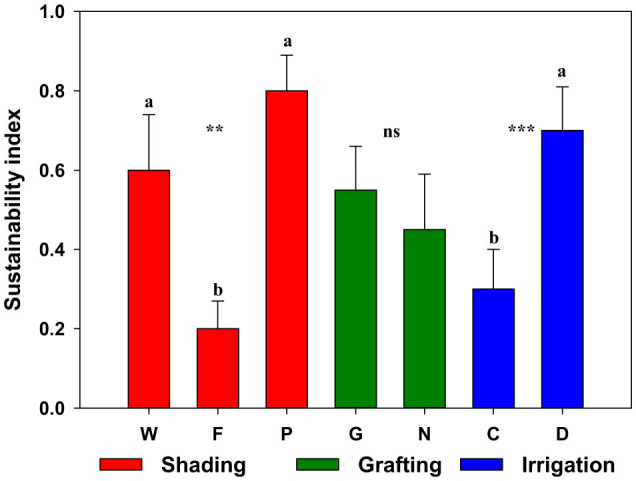
Mean values and standard deviation of the sustainability index for the main factors used (shading, grafting, and irrigation). In each shading type, *n* = 4, W: no shading; F: conventional fixed shading; P: photovoltaic shading. In grafting, *n* = 6, G: grafted plants; N: non-grafted plants. In irrigation, *n* = 6, C: full irrigation; D: deficit irrigation. The differences were analyzed with Fisher's least significant difference test (LSD; *p* = 0.05); different letters in each column indicate significant differences between treatments at *p* < 0.05. In the ANOVA, the significance level is represented by *p* < 0.01 and 0.001 (** and ***, respectively) and “NS” indicates no significant differences.

The analysis of the sustainability of the agroecological strategies used in Muchamiel tomato cultivation, obtained from the IPOWA-CRITIC ranking, offers very consistent results. To improve the interpretation of the results, Figure 2 shows the average allocation values obtained for each of the main factors used. The sustainability of the agronomic strategies is inversely proportional to the values obtained.

The Muchamiel tomato is a crop that is well adapted to Mediterranean edaphoclimatic conditions (Garcia-Martínez, 2016). Thus, the values of commercial production in the treatments without shading are adequate. The plants with fixed and conventional shading had lower production values, perhaps due to reduced photoassimilates. However, their nutritional composition was the best in all the treatments applied. This effect coincides with what was described by Milenkovic et al. (2020); in our experimental conditions, the shading nets were not beneficial for Muchamiel tomatoes. Despite the tomato plants using diffused light more efficiently than direct radiation (Hemming et al., 2008), in our experimental conditions, photoassimilation was limited, negatively affecting production and the index of maturity. It is possible that the reduction in solar radiation caused by the shading nets used was excessive. Milenkovic et al. (2020) used shading nets of different colors, with a reduction in solar radiation similar to that recorded in our study, with tomatoes Optima' F1 and “Big beef” F1, finding an improvement in production and quality. These results can be explained, considering the influence of the genetic material on the response of these plants to these types of treatments. In addition, the color nets have an influence not only on the quantity of solar radiation that reaches the plants but also on their quality (Timmermans et al., 2020).

The shading treatments showed significant differences considering the sustainability criteria used. Thus, the sustainability of the Muchamiel tomato crop without shading and with mobile photovoltaic shading was similar between them, and was higher than the plants with conventional fixed shading (Figure 2). This is because the 50% fixed shade used in the assay limited the photosynthetic activity of the plants, reducing production. Among the no-shade treatments, only the non-grafted plants with full irrigation obtained an unfavorable score (treatment A_3_ occupies 10^th^ place, Table 11). Independent of the shading used, this result can be generalized, that is, the non-grafted plants and those with full irrigation had the worst behavior (treatments A_3_, A_7_, and A_11_ occupied the 10^th^, 12^th^, and 6^th^ positions, respectively, Table 11). The results can be explained if we take into account the effect of the graft on production. Non-grafted plants had a lower production, perhaps due to the presence of fungi and nematodes in the soil, which reduce the absorption of water and nutrients (Phani et al., 2024). Intensive production to maximize performance and satisfy demand makes attacks by plagues and diseases critical threats for producers, in both field conditions and greenhouses (Capinera, 2020; Phani et al., 2021). Despite the heavy losses due to the action of nematodes, the management options of this disease are limited, highlighting grafting among them (Martínez-Ballesta et al., 2010). Therefore, the full irrigation of non-grafted plants implies higher operational costs, although the production is lower than that of the treatments that used grafted plants, so the use of non-grafted plants is particularly unfavorable in our experimental conditions.

**Table 11:** Ranking alternatives.

**Treatment**	**CRITIC**	**TOPSIS**	**VIKOR**	**SAW**	**IPOWA CRITIC**	**IPOWA-S- CRITIC**	**IPOWA-W- CRITIC**	**IPOWA-S-W- CRITIC**
A_1_	5	2	2	6	7	2	9	2
A_2_	1	1	1	4	2	1	2	1
A_3_	10	12	8	8	10	4	10	8
A_4_	4	9	3	7	4	3	4	3
A_5_	11	10	12	12	11	11	11	11
A_6_	8	8	9	9	8	9	6	9
A_7_	12	11	11	11	12	12	12	12
A_8_	9	7	10	10	9	10	8	10
A_9_	6	4	6	2	5	6	5	6
A_10_	3	5	7	5	3	7	3	5
A_11_	7	6	5	3	6	8	7	7
A_12_	2	3	4	1	1	5	1	4
Spearman (*r*_*s*_)	97%	76%	78%	85%		64%	97%	83%

The use of grafts tends to improve the sustainability of the production, considering the criteria utilized (Figure 2). Thus, the evaluation of the grafted plants was better than that of non-grafted ones, in every case, with the exception of the plants under photovoltaic shading and deficit irrigation treatment (treatments A_10_ and A_12_, Table 11). This exception is particularly notable, as treatment A_12_ (PND) was found in first place (Table 11). This result can be explained by considering the multicriteria analysis performed. In this way, the use of deficit irrigation, along with the mobile photovoltaic mesh, mitigates the reduction of production, which implies the use of non-grafted plants in the experimental conditions utilized. On the one hand, the appropriate deficit irrigation of tomatoes can save a significant amount of water and improve quality, without negatively affecting production or the economic results (Khapte et al., 2019). On the other hand, the shade produced by the photovoltaic installation can avoid the reduction in tomato production (Ureña-Sánchez et al., 2012). This could be true, especially in our case, due to the use of the mobile mesh, which only produces shade in the middle of the day. In this way, the photoinhibition of tomato could be avoided, which is produced by excessive radiation, and can lead to a reduction in photosynthetic activity and production (Shi et al., 2022; Wang et al., 2018).

Tomato crops demand a large amount of water (Peet, 2005), especially during the flowering phase (Khapte et al., 2019). Deficit irrigation in the experimental conditions utilized improved the sustainability of the Muchamiel tomato. Thus, the top four treatments used deficit irrigation, and in general, all the even-numbered treatments (deficit irrigation) were better evaluated, that is, their allocation is lower than the odd-numbered treatments just before (identical treatment with full irrigation) (Table 11). Irrigation is a determining factor in the sustainability of production. In areas with warm and/or Mediterranean climates, with a scarcity of water, the maximization of the productivity of water of crops can be more beneficial for the farmer than the maximization of crop performance (Pereira et al., 2002).

Currently, the Overall Life Cycle Sustainability Assessment (OLCSA) is the most utilized method to estimate the sustainability of products, goods, or services. The application to agri-food systems has specific limitations related to the definition of the system, the interval of assessment, or the spatiotemporal resolution of the databases utilized, among others. In addition, there is a potentially high variability between independent agricultural businesses due to the differences on cultivation practices, agroclimatic conditions, seasonality, and distances between the places where activities considered in the lifecycle of the product are carried out (Notarnicola et al., 2017). To avoid these inconveniences, the application of multicriteria methods has been recently developed, such as the one proposed in the present study, which seeks to organize the different strategies as a function of the environmental, economic, social, cultural, etc., criteria. Thus, studies have been conducted on the valorization of agricultural waste (Escalante et al., 2016), joint management of agro-livestock farms and agricultural farms (Reyna-Ramírez et al., 2025), tomato ketchup packaging design (Wohnera et al., 2020), irrigation management in conditions of water deficit (Montazar and Snyder, 2012), fertigation management in tomato cultivation (Heiba et al., 2023), strategies for improving field-grown cereal yield (Di Bene et al., 2022), and evaluation of the sustainability of tomato cultivation (Sadiq et al., 2025). Overall, this is a very interesting approach and is considered a suitable complement to OLCSA methods for decision-making in the agri-food value chain. The continued performance of this type of analysis is needed to make advances on the sustainability of the agricultural sector, improve its resilience, and respond to societal demands.

## 4 Conclusion

The prioritization of agricultural production processes is an extremely complex process, which on many occasions requires subjectivity from the agents involved in the selection process. The CRITIC methodology is based on the objective information from the results of different treatments. However, the opinion of the decision-makers must be considered, so that the introduction of the OWA allows us to weigh the different attributes so that they can be overestimated or underestimated according to the attitudinal character of the decision-maker.

The present study has proposed an extension of Pearson's correlation coefficient, named Pearson-IPOWA, which allows for the calculation of the correlation, considering the attitudinal character of the decision-maker, weighing to a greater or lesser extent, and the elements that have a sum higher than their relative scores.

The introduction of the IPOWA correlation coefficient, together with the use of the OWA-variance, has allowed us to propose an IPOWA-CRITIC that adequately introduces the attitudinal character of the decision-maker, as well as its extensions IPOWA-S-CRITIC, IPOWA-W-CRITIC, and IPOWA-S-W-CRITIC. Finally, the comparison with the traditional CRITIC method with the diverse alternatives proposed allows us to see how the attitudinal character of the decision-makers affects the final ranking of the treatments.

The results of the classifications conducted indicate that the use of mobile photovoltaic mesh is a sustainable production strategy, due to its effect on production and quality of the crop, CO_2_ fixation, and irrigation water savings.

The proposed methodology for calculating the correlation coefficient and its application in the CRITIC is a generalization of the traditional method. It is evident that the use of different induced variables can lead to differences in the final results, so the result shown can be considered a particular case of all the possibilities offered by the proposed methodology. Therefore, it is essential that in each case, the decision-maker selects the one most appropriate to their objectives and needs. In this case, the selected variable shows a very high degree of correlation with the other methodologies with which it has been compared. It is necessary to continue with this type of analysis to facilitate the making of decision of farmers and to make advances on the sustainability of the processes of agricultural production and the agri-food sector.

## Data Availability

The raw data supporting the conclusions of this article will be made available by the authors, without undue reservation.
